# Supplementary material for: First Application of a Mixed Porcine–Human Repopulated Bioengineered Liver in a Preclinical Model of Post-Resection Liver Failure
Source: Biomedicines. 2024 Jun 7;12(6):1272. doi: 10.3390/biomedicines12061272 (PMC11201206; doi:10.3390/biomedicines12061272)
Supplement: Supplementary file 1 [file biomedicines-12-01272-s001.zip › biomedicines-2965135-supplementary.pdf]

First Application of a Mixed Porcine-Human Repopulated Bioengineered Liver in a Preclinical Model of Post-Resection Liver Failure

Philipp Felgendreff [1,2]†[*], Seyed M. Hosseiniasl[1] †, Anna Minshew[1], Bruce P. Amiot[1], Silvana Wilken[1], Boyukkhanim Ahmadzada[1], Robert C Huebert[3], Nidhi Jalan Sakrikar[3], Noah G Engles[3], Jeffrey J Ross [4], Scott L. Nyberg [1, 5]

Supplementary Materials

Intracranial pressure (ICP) during extracorporeal Therapy (ECT)

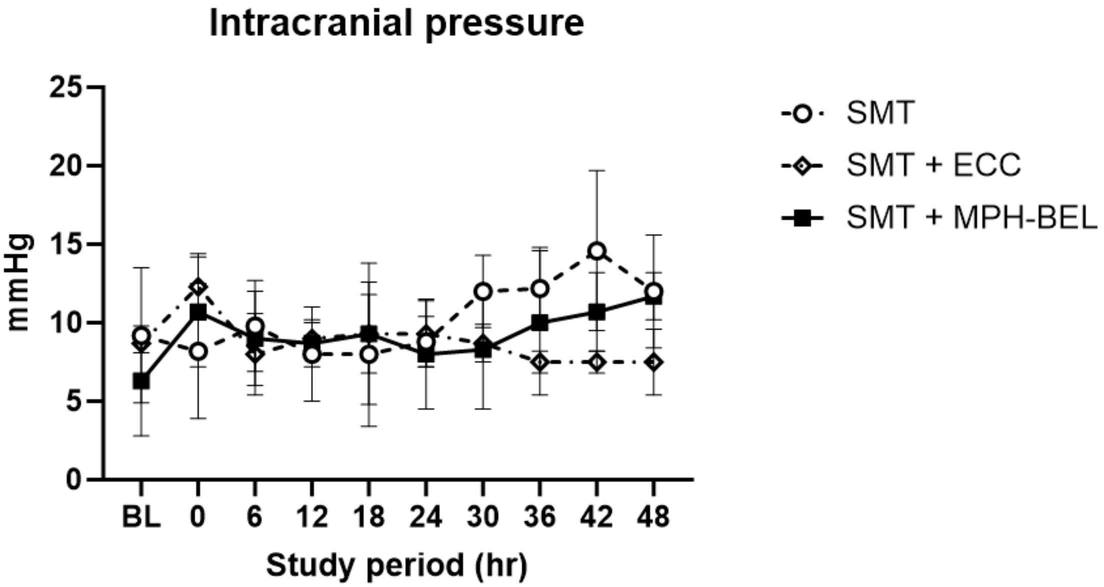

Supplementary Figure S1. ICP during ECT along with the study interval.; SMT: standard medical therapy, SMT+ECC (extracorporeal circuit without BEL graft), SMT+MPH-BEL (Bioengineered livers containing mixed populations of porcine hepatocytes and human endothelial cells).

Biochemical Markers during PRLF

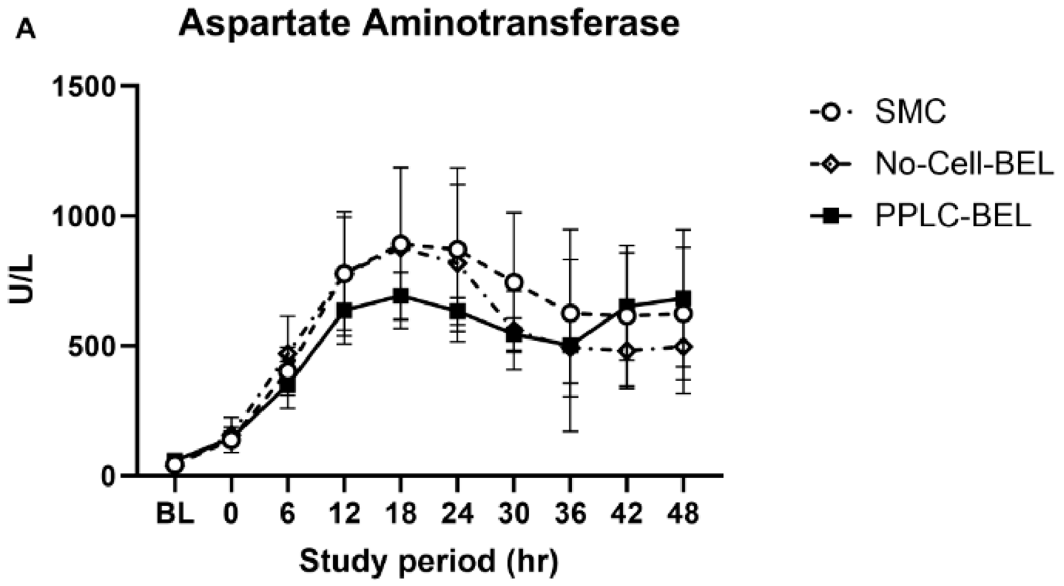

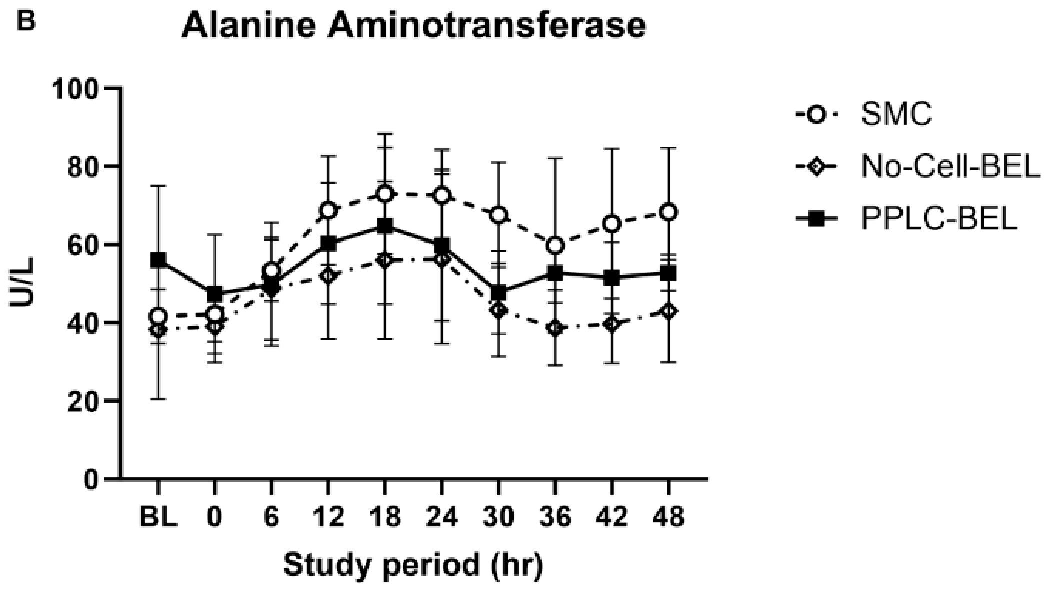

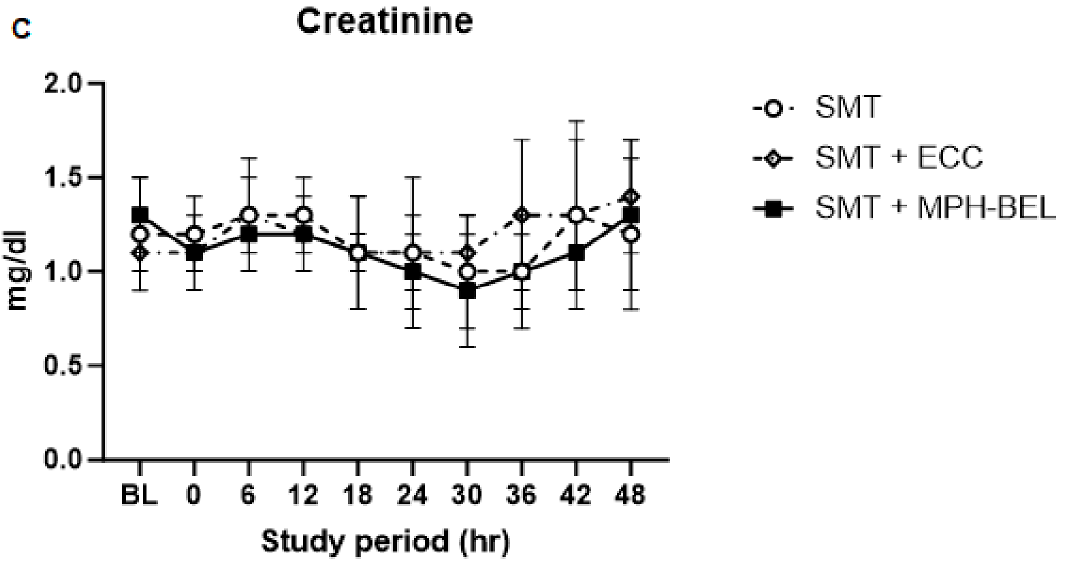

Supplementary Figure S2. Serum concentration of Aspartate Aminotransferase (A), Alanine Aminotransferase (B) and Creatinine in the three study groups (SMT, ECC-group and MPH-BEL) along with the observation period.; SMT: standard medical therapy, SMT+ECC (extracorporeal circuit without BEL graft), SMT+MPH-BEL (Bioengineered livers containing mixed populations of porcine hepatocytes and human endothelial cells).
